# Primary gastric lymphoma in a soldier presenting as acute gastrointestinal bleeding

**DOI:** 10.1186/2054-314X-1-4

**Published:** 2015-01-27

**Authors:** Ishay Ostfeld, Roei Hod Feins, Ory Rouvio, Lev Dorfman, Jacob Moran-Gilad

**Affiliations:** 10 Israeli Defense Forces Medical Corps, Tel-Hashomer, Israel; 11 Hematology Institute, Soroka University Medical Center, Beer-Sheva, Israel; 12 Public Health Services, Ministry of Health, 39 Yermiyahu St, Jerusalem, Israel

**Keywords:** Lymphoma, Gastrointenstinal, Soldier, Hemorrhage

## Abstract

Acute epigastric pain is commonly encountered among young adults undergoing military training. Gastric malignancy usually affects older individuals and may occasionally masquerade as peptic disease. We report a case of primary gastric lymphoma (PGL) in a young soldier, presenting as an acute upper gastrointestinal tract bleeding. The case is presented along with a review of the relevant literature. Primary care physicians should bear in mind that although highly unusual in this age group, primary gastric lymphoma may occasionally afflict young adults and military recruits as demonstrated by the case under discussion. Nevertheless, referral for investigation of suspected peptic disease should follow standard guidelines.

## Background

Acute epigastric pain is commonly encountered among young adults undergoing military training. Gastritis, peptic disease (gastric or duodenal ulcers), gastroesophageal reflux disease, drug adverse effects and functional dyspepsia are all common causes for epigastric pain in this age group. Gastric malignancy usually affects older individuals and may occasionally masquerade as peptic disease. We report a case of primary gastric lymphoma (PGL) in a young soldier, presenting as an acute upper gastrointestinal tract bleeding. The case is presented along with a review of the relevant literature.

## Case presentation

An 18-year-old male soldier was referred to a basic training clinic for evaluation of an epigastric pain. The soldier underwent a standard medical evaluation several months prior to recruitment which was unremarkable. In the office, the soldier reported having been treated several weeks pre-recruitment with a standard eradication regimen for presumed *H. pylori* infection diagnosed by both serology and urease breath test. Endoscopy was not performed.

The soldier reported a constant, stabbing, non-radiating epigastric pain accompanied by nausea. No aggravating or alleviating factors were identified. He denied having fever, vomiting, diarrhea, bloody stools, melena or weight loss. Upon physical examination, left epigastric tenderness was noticed on abdominal palpation. The rest of the examination was unremarkable. The soldier was prescribed oral H2 blockers with a working diagnosis of peptic disease and was referred to a gastroenterologist for further evaluation.

Three days later, the soldier was urgently referred back to the clinic due to a worsened abdominal pain, and marked fatigue. Physical examination revealed a heart rate of 120/min, blood pressure 120/75 mmHg and oral temperature of 37°c. Abdominal examination revealed only mild epigastric tenderness. During observation, hypotension which responded to volume expansion was evident and the patient was thus urgently evacuated to the nearest hospital.

Upon hospital admission gastric contents resembling 'Coffee Ground’ were identified following nasogastric intubation and a rectal examination revealed typical melena. Laboratory tests revealed severe acute blood loss with a hemoglobin of 4.5 gr%, hematocrit 14%, normocytic red blood cell indices, mild leukocytosis and notable thrombocytosis. Reticulocytosis was noted on peripheral blood smear. Emergency gastroscopy revealed a large ulcerated gastric mass in the gastric antrum which was biopsied. The gastric bleeding ceased spontaneously without a need for any additional intervention.

**Figure 1:**
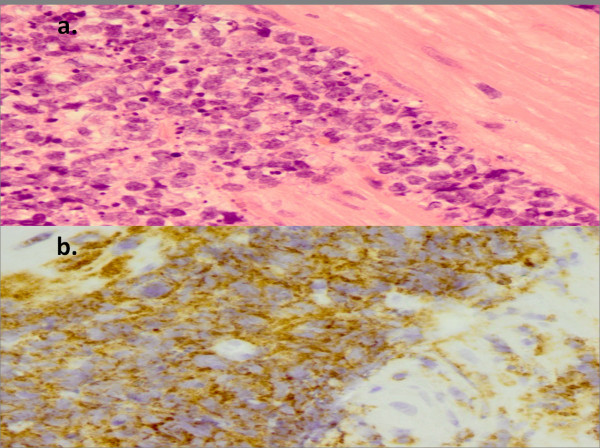
**Histopathological findings.** H&E staining of the gastric biopsy revealed a large cell lymphoma infiltrate **(1a)**. CD20 staining highlights a diffuse infiltrate of neoplastic B cells **(1b)** histopathological findings from gastric biopsy.

**Figure 2:**
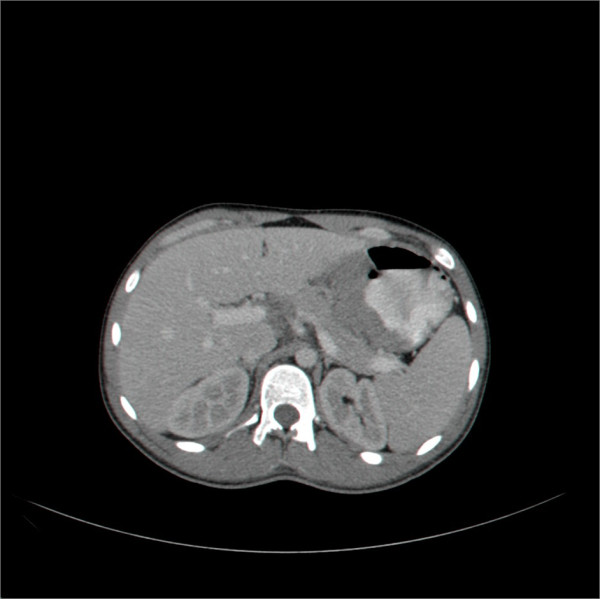
**Findings in abdominal computed tomography consisting of a gastric mass.**

Histopathological examination revealed a B-cell lymphoma; tumor cells were large and positive for LCA, CD20, CD79a with Ki67+ (95-100%), consistent with an aggressive diffuse large B-cell lymphoma, with very high proliferation index and apoptosis (Figure 1). Abdominal computed tomography demonstrated marked concentric thickening of gastric antrum with luminal narrowing. The liver was homogenous and no lymphatic involvement was observed (Figure 2). No evidence for thoracic involvement was noted on chest CT. Primary gastric diffuse large B-cell lymphoma (PG-DLBCL) stage IE was thus diagnosed. Bone marrow biopsy revealed no infiltration.

Following standard protocols for pre-medication, cardiac evaluation and sperm cryopreservation, chemotherapy consisting of intravenous rituximab (8 cycles) in combination with CHOP (6 cycles) was initiated. Adjuvant pegylated G-CSF was administered following each cycle and no significant complications were noted. PET-CT evaluation after 4 R-CHOP cycles revealed complete resolution of the gastric lymphoma mass without any FDG uptake. A repeated PET-CT after conclusion of chemotherapy showed normal anatomy without any FDG uptake or evidence for active lymphoma. The patient was relieved from military duty and volunteered to service 6 month later upon remission. The patient is in complete remission over 2.5 years of follow-up.

## Conclusions

Primary gastric diffuse large B-cell lymphoma (PG-DLBCL) is a part of a larger group termed primary extra-nodal non-Hodgkin’s lymphomas (PE-NHL). The term PE-NHL refers to lymphomas which present with disease at any organ or tissue other than the lymph nodes or the spleen; the symptoms at initial presentation are caused mainly by extra-nodal involvement and after routine staging procedures, the extra-nodal involvement remains the clinically dominant site of the disease.

PE-NHL comprise ∼ 25–40% of NHL, while primary NHL of the gastrointestinal tract is the most commonly involved extra-nodal site and represents 10–15% of all NHL cases and 30–40% of all extra-nodal sites. The most commonly involved site is the stomach (60–75% of cases), followed by the small bowel, ileum, cecum, colon and rectum [1]. All histological categories of nodal lymphomas may arise in the gastrointestinal (GI), but the main two histological subtypes accounting for over 90% of cases are mucosa-associated lymphoid tissue (MALT) NHL and diffuse large B-cell (DLBCL) NHL.

The diagnosis of primary gastric lymphoma relies on esophagogastroduodenoscopy and biopsy, followed by further testing and imaging for staging purposes, including computed tomography, endoscopic ultrasonography, positron emission tomography and bone marrow examination [2]. The treatment of choice for DLBCL irrespective of anatomic site is rituximab combined with an anthracycline-based combination chemotherapy. The role of surgery in the management of PG-DLBCL, particularly gastrectomy, is highly controversial. Radiotherapy may reduce the incidence of relapse but does not affect overall survival. With adequate treatment, prognosis is good, with a 5-year overall survival of nearly 90%.

PG-DLBCL usually affects patient between the 4^th^ and 6^th^ decades of life. In one study, the mean age was 57.7 years [3] while the median age was even higher in another [2]. There is a slight male preponderance [2]. Cases among individuals prior to the second decade of life are unusual. According to the IDF recruits database, only 2 cases of gastric malignancies were reported during the last 5 years, one of them was MALT NHL, the other being a carcinoma.

The clinical presentation of PG-DLCBL is non-specific. The initial symptoms of upper abdominal pain and early satiety may be vague and nonspecific. Other common symptoms include weight loss, nausea, vomiting, abdominal fullness and indigestion. Symptoms and signs may mimic those of other abdominal pathologies which are prevalent among young adults, including infectious gastroenteritis, functional dyspepsia, gall bladder disease, and peptic ulcer disease. Weakness, night sweats, jaundice, fever and dysphagia occur less frequently. Young adults, and especially soldiers, in whom there is a low index of suspicion for gastric malignancy may suffer a delayed diagnosis. Dietary changes, low hygiene conditions and physical and emotional stress in the military setting could further complicate the diagnosis.

Occasional patients present with covert bleeding (as in our patient), or in the form of frank hematemesis or melena, while gastric obstruction and perforation are less common. Uncommon complications include gastrocolic fistula, gastrointestinal and intra-abdominal hemorrhage resulting from invasion of a primary gastric lymphoma into the spleen [4]. In a series of low and high-grade primary gastric lymphomas the rate of GI bleeding as a presenting symptom was 15.8% [3]. In another series, hematemesis and melena were reported among 13.6% and 12.9% of patients with low- and high-grade gastric lymphoma respectively. Notably, gastrointenstinal bleeding comprised 32% of 'alarm signs’ reported [5].

The approach to dyspepsia in the young adult age group (<50 years) involves an investigation of common causes such as gastritis, peptic disease (gastric or duodenal ulcers), gastroesophageal reflux disease, drug adverse effects and functional dyspepsia. Uncommon causes may include neoplasms and cardiovascular conditions. In the absence of 'red flags’ such as unexplained weight loss, recurrent vomiting, progressive dysphagia, odynophagia, gastrointestinal blood loss or family history of upper GI cancer the common disorders listed above should be investigated [6]. These should include discontinuation of medications known to cause dyspepsia. Predominant reflux symptoms warrant a therapeutic trial with proton pump inhibitors while predominant dyspepsia without reflux warrants a work up for *H. pylori* and appropriate therapy based on the results. Endoscopic studies are usually reserved for cases refractory to standard therapy with proton-pump inhibitors and/or antimicrobial agents or for those with unexplained etiology or 'red flags’.

Primary care physicians should bear in mind that although highly unusual in this age group, malignant neoplasm may occasionally afflict young adults and military recruits as demonstrated by the case under discussion and prompt referral for investigation may prevent further delays in diagnosis. Nevertheless, referral for investigation of suspected peptic disease should usually follow standard guidelines.

## Consent

Written informed consent was obtained from the patient for publication of this case report and any accompanying images. A copy of the written consent is available for review by the Editor-in-Chief of this journal.

## Abbreviations

NHL: Non Hodgkin’s lymphoma
PGL: Primary gastric lymphoma
DLBCL: Diffuse large B-cell lymphoma
G-CSF: Granulocyte colony stimulating factor
CT: Computerized tomography
PET: Positron emission tomography.
